# Comparison of Tobacco Control Scenarios: Quantifying Estimates of Long-Term Health Impact Using the DYNAMO-HIA Modeling Tool

**DOI:** 10.1371/journal.pone.0032363

**Published:** 2012-02-23

**Authors:** Margarete C. Kulik, Wilma J. Nusselder, Hendriek C. Boshuizen, Stefan K. Lhachimi, Esteve Fernández, Paolo Baili, Kathleen Bennett, Johan P. Mackenbach, H. A. Smit

**Affiliations:** 1 Department of Public Health, Erasmus MC, University Medical Center Rotterdam, Rotterdam, The Netherlands; 2 Centre for Prevention and Health Services Research (PZO), National Institute for Public Health and the Environment (RIVM), Bilthoven, The Netherlands; 3 Department of Statistics and Mathematical Modeling, National Institute for Public Health and the Environment (RIVM), Bilthoven, The Netherlands; 4 Department of Human Nutrition, Wageningen University, Wageningen, The Netherlands; 5 Tobacco Control Unit, Institut Català d'Oncologia-IDIBELL, L'Hospitalet de Llobregat (Barcelona), Barcelona, Spain; 6 Department of Clinical Sciences, School of Medicine, Campus of Bellvitge, Universitat de Barcelona, Barcelona, Spain; 7 Descriptive Studies and Health Planning Unit, Fondazione IRCCS “Istituto Nazionale Tumori”, Milan, Italy; 8 Department of Pharmacology & Therapeutics, Trinity Centre for Health Sciences, St James's Hospital, Dublin, Ireland; 9 Julius Center for Health Sciences and Primary Care, University Medical Centre Utrecht, Utrecht, The Netherlands; INSERM & Universite Pierre et Marie Curie, France

## Abstract

**Background:**

There are several types of tobacco control interventions/policies which can change future smoking exposure. The most basic intervention types are 1) smoking cessation interventions 2) preventing smoking initiation and 3) implementation of a nationwide policy affecting quitters and starters simultaneously. The possibility for dynamic quantification of such different interventions is key for comparing the timing and size of their effects.

**Methods and Results:**

We developed a software tool, DYNAMO-HIA, which allows for a quantitative comparison of the health impact of different policy scenarios. We illustrate the outcomes of the tool for the three typical types of tobacco control interventions if these were applied in the Netherlands. The tool was used to model the effects of different types of smoking interventions on future smoking prevalence and on health outcomes, comparing these three scenarios with the business-as-usual scenario. The necessary data input was obtained from the DYNAMO-HIA database which was assembled as part of this project. All smoking interventions will be effective in the long run. The population-wide strategy will be most effective in both the short and long term. The smoking cessation scenario will be second-most effective in the short run, though in the long run the smoking initiation scenario will become almost as effective. Interventions aimed at preventing the initiation of smoking need a long time horizon to become manifest in terms of health effects. The outcomes strongly depend on the groups targeted by the intervention.

**Conclusion:**

We calculated how much more effective the population-wide strategy is, in both the short and long term, compared to quit smoking interventions and measures aimed at preventing the initiation of smoking. By allowing a great variety of user-specified choices, the DYNAMO-HIA tool is a powerful instrument by which the consequences of different tobacco control policies and interventions can be assessed.

## Introduction

After half a century of tobacco control policy, a vast range of interventions has been proposed, evaluated and implemented with varying degrees of success, though none of these have turned out to be fully effective in the worldwide eradication of tobacco consumption as a deadly habit [1], [2], [3]. In the Netherlands overall smoking prevalence is still high at 27% and has remained relatively constant over the past decade. Among adolescents 21% declared to be smoking in 2010 [4]. Policymakers are required to choose which of the numerous interventions to implement, but lack quantitative information on the long term impact of such interventions on population health. Would it be more effective to target smoking interventions to stimulate smokers to quit, or to discourage adolescents from initiating smoking, or should policy measures be targeted population-wide by advertisement restrictions, smoke-free public places or tobacco price adjustments? And how will this affect the smoking distribution and population health in the short and long term? Existing Dutch tobacco control policies and interventions currently include some in-school smoking prevention interventions for those aged 10 to 12 and smoking cessation interventions for adults. The latter mainly consist of telephone help lines, intensive telephone coaching, and tailored online quit smoking advice. Regarding population-wide tobacco control policies the Netherlands has implemented advertising restrictions, health warnings and smoke-free legislation, but there is potential for even more stringent legislation like a further tax increases, as currently the tax percentage of the retail price of cigarettes is still below the officially recommended level [3].

Interventions differ in terms of their effectiveness and their ability to reach different population groups. All vary in terms of efforts and implementation costs [5]. Changing demographic characteristics, competing morbidity as well as age-dependent patterns of disease incidence, mortality and relative risks (RRs) associated with smoking complicate the possibilities to quickly oversee the consequences of tobacco control scenarios on future population health, and hamper informed decision making.

We developed a software tool, DYNAMO-HIA, which allowed us to make a quantitative comparison of the health impact of different policy scenarios over time, by comparing the policy scenario with the “business-as-usual” scenario, i.e. no change as compared to the current situation. The tool has been described in more detail elsewhere [6]. Here we will illustrate the capacities of the DYNAMO-HIA model to estimate the long term health impact of three typical types of tobacco control interventions if these were applied in the Netherlands, alluding to Rose's distinction between high-risk vs. population wide approaches [7]. We concentrate on the following interventions: 1) smoking cessation interventions in adult smokers 2) preventing smoking initiation in adolescents and 3) implementation of a nationwide intervention affecting quitters and starters simultaneously, by adjusting the price of cigarettes through increased taxation. Using these three scenarios, we demonstrate the possibilities to dynamically quantify notions which are known intuitively. To measure the impact on health we focus on the future prevalence of smoking-related chronic diseases such as lung cancer, chronic obstructive pulmonary disease (COPD) and ischemic heart disease (IHD), as well as on mortality.

## Methods

### Description of DYNAMO-HIA

DYNAMO-HIA is a recently-developed Markov type, multi-state simulation software. It was developed to allow researchers and policy makers in the field of Health Impact Assessment (HIA) to 1) quantify the development of risk factor exposure over time and to 2) estimate the impact of these changes in risk factor exposure on disease prevalence, mortality and on summary measures of population health. DYNAMO-HIA is a dynamic tool that synthesizes data according to the causal epidemiological pathway, linking risk factor exposure through relative risks (RRs) of incidence of associated diseases and death, to prevalence of diseases, mortality and summary measures of population health, and allowing to take into account relative risks by “time since quitting smoking” and age, as well as competing risks. Following the epidemiological causal chain implies that the model uses relative risks by risk factor class, i.e. incidence in exposed risk factor classes are a multiple of the incidence in the non-exposed. A change in risk factor exposure due to the policy or intervention thus changes disease incidence and in turn disease prevalence and mortality. The effect of the risk factor change on mortality through diseases not included in the model, i.e. other-cause mortality, is taken into account by additionally using the relative risk on total, i.e. all-cause, mortality. Other mortality is derived from total mortality and disease specific mortality, assuming additive mortality [8].

In order to isolate the effects of the intervention DYNAMO-HIA always compares one or more intervention scenarios which result in a modified risk factor prevalence and/or modified transition rates, with the reference or business-as-usual scenario.

DYNAMO-HIA requires input such as 1) demographic data, including population numbers, numbers of future newborns and all-cause mortality, and 2) epidemiological information on incidence, prevalence and mortality (IPM) for relevant diseases, risk factor exposure, as well as relative risks linking exposure to disease and to all-cause mortality, all by age and sex. The present version of the DYNAMO-HIA software package, which is publicly available at: www.dynamo-hia.eu, includes input data on risk factor prevalence, relative risks, and IPM information for nine diseases for a large set of EU member states. The diseases included in the model are diabetes, ischemic heart disease, stroke, lung cancer, oral cancer, esophageal cancer, colorectal cancer, breast cancer and COPD. The risk factors include the body mass index (BMI), alcohol and tobacco consumption. Time since quitting smoking is taken into account by including prevalence and relative risks in former smokers by time since quitting. The model provides output on summary measures of population health such as life expectancy with and without disease, mortality, survival as well as disease and risk factor prevalence by age and sex. The effect of an intervention or policy on future risk factor exposure and future health is assessed by comparing one or more scenarios with a specific intervention or policy change to a scenario without any intervention, so business-as-usual. The effects of the intervention or policy on the risk factor prevalence in the first year and/or on transitions between risk factor states (i.e. smoking (re) start and quit rates) are given by the user. The risk factor prevalence in future years is an outcome of the model. The theoretical specifications of the model have been described elsewhere [6].

### Three smoking intervention scenarios and a reference scenario

We evaluated the effects of three intervention scenarios, each reflecting one of the three basic types of tobacco control: 1) interventions to increase quitting, 2) interventions to reduce smoking initiation and 3) policies reducing population-wide smoking. Interventions to increase the quit rate among smokers are usually targeted towards adults and include measures such as counseling and personal or grouped pharmacological and/or psychological therapy. Interventions to decrease or prevent smoking initiation usually target adolescents and are often school-based interventions. Nationwide policy measures for population-wide smoking reduction, such as the use of tobacco price taxation, affect quitting and starting simultaneously.

Each intervention scenario is characterized by a change in smoking prevalence in the first year, i.e. just after the intervention or policy, and/or by changed (re)start and quit rates, as compared to the reference scenario. In addition, the proportion of the target population that will effectively be reached by the intervention characterizes the intervention scenario. We modeled both a maximum scenario, which gives a better impression of the varying effects over age and time for maximally effective interventions, versus a more realistic scenario version. To quantify the order of magnitude of the change in smoking prevalence and/or (re)start and quit rates in the target population, we evaluated systematic reviews/meta-analyses, and where necessary, primary articles of intervention studies, based on a PubMed literature search.

#### Reference scenario

The reference scenario starts from the current prevalence of never, former and current smokers by age and sex, and from current transition probabilities between the risk factor states over the life course. The current prevalence e.g. specifies what percentage of those presently 20 years old are never, former or current smokers and the current transition rates, i.e. (re)start and quit rates of smoking, specify how many of the currently 20 years old never smokers will remain never smokers when they are 21, 22 etc. years old, and how many start smoking when they are 21, 22 etc. years old. The current prevalence and transition rates relate to the business-as-usual situation, that is, a situation with smoking control measures that are already in place, but without the specific intervention. Dutch baseline prevalence of smokers, former smokers and never smokers and smoking (re)start and quit rates used here are included as supporting information (Appendix S1). The DYNAMO-HIA database provided information on smoking prevalence, i.e. the percentage of current smokers, former smokers and never smokers for ages 16 and over, based on the POLS study [9] (for further information please refer to the data documentation section of the DYNAMO-HIA project website: www.dynamo-hia.eu). Smoking prevalence, i.e. the percentage of smokers and the percentage of non-smokers for ages 10 to 15 was derived from Stivoro's Jeugdmonitor (Youth monitor) [10], which is the Dutch center for expertise on tobacco prevention. The age- and sex specific start, quit and restart rates for ages 16 years and over were also based on information available through Stivoro [11]. For ages up to age 16, “net” smoking initiation rates were estimated using a standard life table of a cohort of non-smokers, whose number decreases with age because persons take up smoking. Using net initiation rates means that flows into the non-smoking state are not explicitly modeled, e.g. if 100 adolescents start smoking and 4 quit, the net uptake is 96. Also restart rates are not separately modeled at these ages.

Relative risks from smoking categories to diseases and all-cause mortality used in this analysis as well as an overview of the age-specific disease prevalence at baseline are also included as supporting information (Appendix S2).

#### The “smoking cessation intervention”: change in quit behavior

For the first scenario, the “smoking cessation intervention”, we chose an odds ratio (OR) of 2.0 reflecting that the ORs quantifying the effects of interventions on cessation rates varied from 1.4 to 2.2 among persons aged 18 years and over to which these interventions are usually targeted [12], [13], [14], [15], [16]. This resulted in post-intervention cessation rates about twice as large as in the reference scenario. These were assumed to remain constant over the entire projection period.

#### The “smoking initiation intervention”: change in start behavior

For the second scenario, the “smoking initiation intervention”, we assumed a 50% decrease in the smoking initiation rate for those at school ages 10–18 in the maximum scenario, and a 20% reduction in the realistic scenario version, reflecting that the literature showed mixed results varying from no effects to a significant reduction in start rates [17], [18], [19]. These post-intervention initiation rates were assumed to remain constant over the entire projection period.

#### The “population-wide smoking control policy”: change in (re)start and quit behavior

For the third scenario, the “population-wide smoking control policy”, we almost doubled the price of tobacco products. That is, we chose to use a 95% increase in the price of tobacco in the maximum scenario, which reflects the price adjustment if the Netherlands was to increase the price of tobacco to match the price of tobacco in Ireland, which currently has the highest tobacco price in the EU [20]. In the realistic scenario version, we assumed a smaller price increase of 20%.

The effect of the price increase on smoking is based on a price elasticity, which measures the average proportional reduction in demand when the price of a commodity increases. We used a price elasticity of smoking prevalence of −0.4 for persons aged 21 and over and of −0.7 for persons up to age 20, who usually show greater responsiveness [21]. Hence, we assumed that a 95% increase in price in the maximum scenario leads to a 66.5% (i.e. 0.7*95%) reduction in smoking prevalence among persons below age 21, and for persons aged 21 and over to a 38% (i.e. 0.4*95%) reduction. In the realistic scenario, we used 14% and 8%, respectively. Given that most smokers start smoking before age 21, we further assumed that for adults the decrease in prevalence of smokers originates from an increase of former smokers, i.e. higher quit rates and not from lower start rates, and that the adults who quit smoking do so immediately after the price increase, that is, we assumed that they will not show any delayed change in smoking status in the years after the price change. Therefore, we left their future transition probabilities unchanged, except for the restart rates. Restart rates were adjusted by the same percentage as the start rates, based on the assumption that if persons quit because of the higher price, this high price will also reduce their likelihood to restart smoking in the future. These new start rates were assumed to be valid during the whole projection period. For persons up to the age of 20 we assumed that decreases in the prevalence of smoking originate from an increase in never-smokers, i.e., fewer people starting to smoke. We also assumed that children in future years, upon reaching the ages where they would take up smoking, would have the same smoking prevalence as their peers after the intervention. Given the higher price they are assumed to be less likely to start smoking as compared to the situation with the lower price. To ensure that the future prevalence of smoking among adolescents remained at this post-intervention level, starting rates were obtained that are consistent with the new, lower smoking prevalence, also using the above life table approach.

#### Reach of the interventions

In the maximum scenarios, we assumed that 100% of the target population will be reached by the interventions. However, given that the size of the population that will be reached by an intervention is likely to be smaller, and is likely to differ by type of intervention, we assumed a lower reach for the smoking cessation intervention and the smoking initiation intervention in the realistic scenario versions. Considering that approximately 40% of smokers are willing to give up smoking in the coming year [22], and assuming that, due to possible supply-side constraints of such interventions, only about 50% of those wanting to quit will actually participate in the interventions, we used a reach of 20%. In the realistic version of the smoking initiation intervention we assumed that, while virtually all adolescents are at risk of taking up smoking, only half of them will be reached by these school interventions. For the population-wide smoking control policy, we assume that both in the maximum and realistic scenarios virtually the whole population will face the higher price, and hence made no distinction between the reach of the maximum and realistic scenario versions. The assumptions for the maximum and realistic scenario versions are summarized in Table 1.

**Table 1:** Interventions used in this paper, maximum vs. realistic scenarios versions.

Scenario	Maximum scenario versions+		Realistic scenario versions++	
	Impact	Reach	Impact	Reach
1. “Smoking Cessation Intervention”Targeting adult smokers (18 yrs and over) to quit through quit intervention	OR: 2.00 on quit rate	100% of smokers	OR: 2.00 on quit rate	20% (40% smokers want to quit * 50% of those are reached)
2. “Smoking Initiation Intervention”Targeting adolescents (10–18 yrs) not to start through an in-school intervention	50% decrease of start rate	100% of non-smokers	20% decrease of start rate	50% (100% at risk to start, 50% reached)
3. “Population-wide Smoking Control Policy”Targeting entire population through a price increase (95% in max. and 20% realistic scenario)	Ages up to 20: increase never smokers by 66.5% and reduction start rates. Ages 21 and above: increase former smokers by 38%. Decrease of restart rate to 30% of reference.	100% of entire population	Ages up to 20: increase never smokers 14% and reduction start rates. Ages 21 and above: increase former smokers 8%. Decrease of restart rate to 80% of reference.	100% of entire population

**Figure 1:**
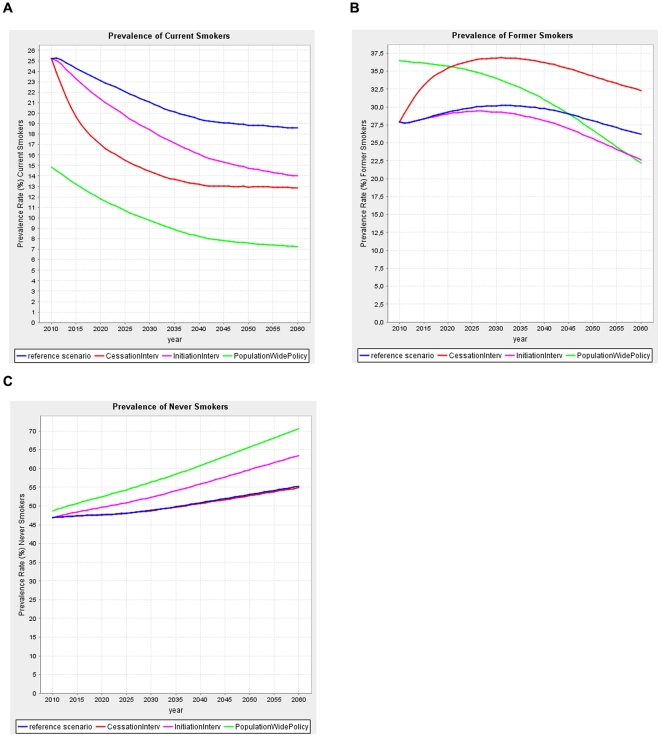
Smoking prevalence over time; Effects of each scenario in the Netherlands (maximum version, panels a–c).

**Figure 2:**
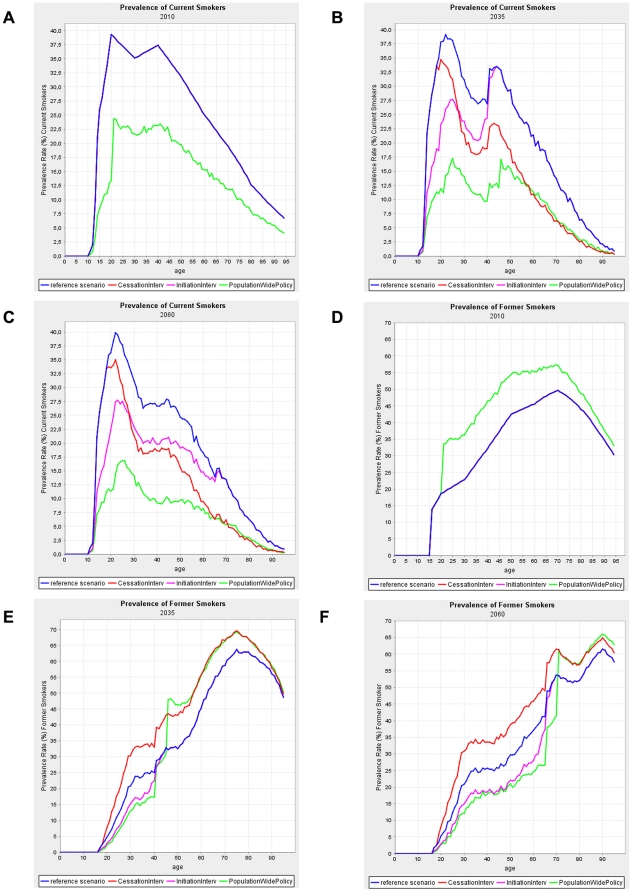
Smoking prevalence by age; Effects of each scenario in the Netherlands (maximum version, panels a–f).

**Figure 3:**
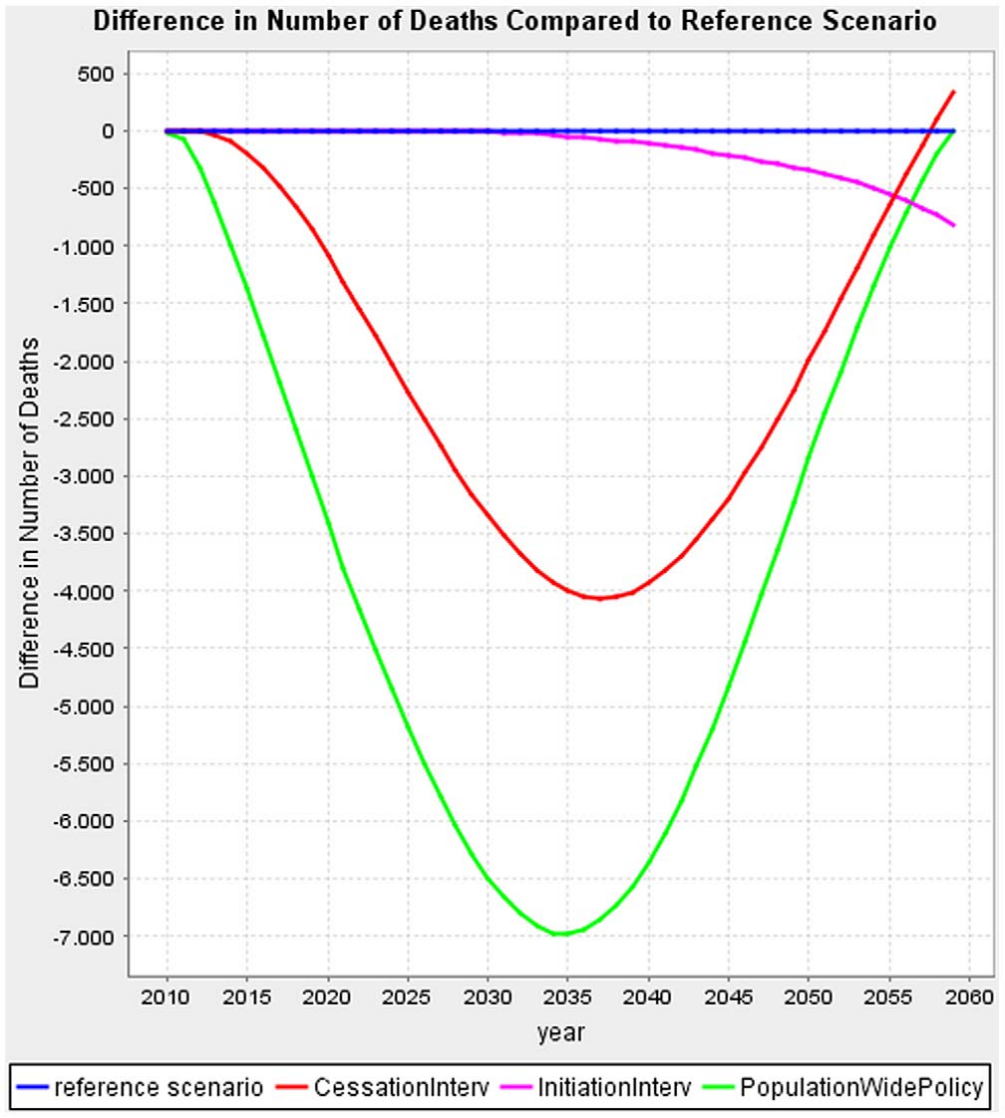
Difference in the number of deaths; Effects of each scenario in the Netherlands (maximum version).

+ See figures 1a–c, 2a–f and 3, table 2 for results.

++ See figures S1a–c, S2a–f and S3, table S1 for results.

We compared the changing patterns of smoking prevalence and health impact of each of these scenarios with the reference scenario over time, using DYNAMO-HIA.

## Results

### Effect of interventions on future prevalence of current, former and never smokers

The smoking cessation intervention will cause an initially strong decrease in the prevalence of current smokers, mirrored by an increase in the prevalence of former smokers as compared to the reference scenario (Figure 1a–c). By definition the prevalence of never smokers is not affected by this intervention. In the first years after the intervention, the prevalence of current smokers decreases more quickly than in the reference scenario, yielding an increasing reduction in the prevalence of current smokers due to the intervention. After 15 years (year 2025), this reduction in the prevalence of current and former smokers becomes stable. In the smoking cessation scenario the prevalence of current smokers is estimated to fall to 14% in 2035, versus 20% in the reference scenario.

The smoking initiation intervention also causes a decrease in the prevalence of current smokers as compared to the reference scenario, but it is smaller than in the smoking cessation scenario. In the short-term this decrease is mirrored by a similar increase in the prevalence of never smokers and no change in the prevalence of former smokers (Figure 1a–c). The overall prevalence of current smokers decreases steadily and more rapidly than in the reference scenario, causing a major change in the age-distribution of current smokers over time (Figure 2a–f). Initially the reduction in smoking prevalence rates due to the intervention only occurs at younger ages. Increased projection time allows the effects to expand to older ages as the adolescents affected by the intervention reach adulthood and in the end old age.

Compared to the reference scenario, the population-wide smoking control policy causes an immediate decrease in the prevalence of current smokers, reflecting that the price increase is assumed to affect behavior virtually immediately. Evidently, this decrease is initially accompanied by a higher prevalence of former and never smokers, as in the model adults were assumed to quit and adolescents not to take up smoking in response to the price increase. Further, in the longer run the prevalence of former smokers becomes lower than in the reference scenario (Figure 1a–c), reflecting that less smoking initiation reduces smoking prevalence and in turn reduces former smoking prevalence. The population-wide smoking control policy affects the prevalence of current smokers in all age groups. Initially an increase of former smokers is seen at all ages, reflecting the massive number of individuals quitting due to the doubling of the price. With increasing projection time the prevalence of former smokers drops below their prevalence in the reference scenario. This effect starts at the youngest ages and with time expands to older ages (Figure 2a–f). This pattern is the net effect of two opposing effects. Firstly, an immediate increase in prevalence of former smokers due to the price increase, and secondly, a delayed opposite effect reflecting that less smoking initiation reduces current smoking prevalence and in turn reduces the prevalence of former smokers. This latter effect expands gradually to older ages.

The set of realistic scenario versions for each of the three types of interventions/policy models smaller effects on smoking exposure. This either reflects less dramatic interventions (e.g. smaller price increase), smaller effects of the interventions on the persons who participate (e.g. 20% reduction in start rates as compared to 50%) and/or a smaller percentage of the target population that participates (reach). This revealed similar patterns of smoking prevalence, though being less pronounced (Figures S1a–c and S2a–f).

### Effect of interventions on future disease prevalence

Table 2 shows the effects of the maximum scenarios on the point prevalence of smoking-related diseases such as lung cancer, COPD, IHD as well as on the prevalence of those with at least one disease, i.e. diabetes, ischemic heart disease, stroke, lung cancer, oral cancer, esophageal cancer, colorectal cancer, breast cancer and COPD for the years 2035 and 2060. On the left we display the absolute baseline level and percentage and the difference due to the intervention in the prevalence of the respective diseases after 25 years, as compared to the reference. On the right we show results after 50 years, i.e. for the year 2060. These are two snapshots in time showing how the effects build up over 25 and 50 years, respectively. Figures showing the evolution over time in more detail are available from the authors on request.

**Table 2:** Effects of scenarios on point prevalence of diseases in the Netherlands (maximum version).

	Absolute Level and Reduction in Disease Prevalence as Compared to Reference Scenario							
	2010–2035				2010–2060			
	Lung Cancer	COPD	IHD	at least one disease	Lung Cancer	COPD	IHD	at least one disease
Absolute Baseline Prevalence 2010	12,863	211,798	508,596	1,483,769	12,863	211,798	508,596	1,483,769
change Scenario 1 (Cessation)	2,957	36,087	23,967	47,712	2,753	39,299	23,684	32,625
change Scenario 2 (Initiation)	3	0	94	118	534	6,167	10,880	17,465
change Scenario 3 (Population-Wide Policy)	5,044	66,952	54,071	92,796	5,006	72,550	63,161	79,467

	Percentage and Reduction in Disease Prevalence as Compared to Reference Scenario in Percentage Points							
	2010–2035				2010–2060			
	Lung Cancer	COPD	IHD	at least one disease	Lung Cancer	COPD	IHD	at least one disease
Baseline Prevalence 2010 in Percent	0.079	1.305	3.130	9.131	0.079	1.305	3.130	9.131
change Scenario 1 (Cessation)	0.018	0.219	0.153	0.314	0.018	0.260	0.179	0.287
change Scenario 2 (Initiation)	0.000	0.000	0.001	0.001	0.003	0.040	0.072	0.118
change Scenario 3 (Population-Wide Policy)	0.030	0.406	0.345	0.620	0.033	0.478	0.453	0.650

*out of: diabetes, ischemic heart disease, stroke, lung cancer, oral cancer, esophageal cancer, colorectal cancer, breast cancer, COPD.

The population-wide smoking control policy causes the largest reduction of the prevalence of lung cancer, COPD, IHD and of persons with at least one disease. By 2035 this intervention prevents about 67,000 COPD cases, 5,000 lung cancer cases, 54,000 IHD cases and about 93,000 cases of persons with at least one disease. The smoking cessation intervention takes a middle position on the prevalence reduction for the listed diseases. The smoking initiation intervention builds up much slower since it will only exert its effects when those prevented from smoking would have otherwise become ill. Thus, even in 25 and 50 years time, the effects on disease prevalence are substantially smaller than in the other two scenarios.

The effects of the realistic scenarios on disease prevalence in 2035 and 2060 were similar in shape, but evidently smaller than in the maximum version. The exact results for the realistic scenario versions can be seen in Table S1.

### Effect of interventions on future deaths/lives saved

Figure 3 shows the difference in the excess number of deaths from all causes by calendar year due to each intervention, the baseline number of deaths being 125,650. The population-wide smoking control policy scenario prevents the most deaths. The effects of the interventions on the excess number of deaths as compared to the reference scenario in the population-wide smoking control policy and the smoking cessation intervention both first show an increase, followed by a reduction. This reflects two opposite effects. Firstly, fewer deaths occur, due to the lower prevalence of smoking, reducing the prevalence of smoking-related diseases. Secondly, more deaths occur in the longer run because the intervention keeps persons alive longer, yielding an on average older population. Simultaneously, this also translates into an increase in healthy life expectancy: HLE for men (women) for maximum scenarios in 2010 at baseline: 68.26 (71.45) years; in 2035 without intervention: 69.70 (71.90), with smoking cessation intervention: 70.25 (72.30), with smoking initiation intervention: 69.71 (71.91) and with population-wide policy: 70.79 (72.73). HLE for men (women) in 2060 without intervention: 70.22 (72.11), with smoking cessation intervention: 70.92 (72.67), with smoking initiation intervention: 70.41 (72.25) and with population-wide policy: 71.64 (73.32). Similar mortality patterns can be observed for the realistic scenario versions, displayed in Figure S3.

## Discussion

### Main findings

The comparison of the three types of interventions shows that the population-wide smoking control policy causes an instant exposure improvement, while also resulting in the largest decrease in the prevalence of current smokers, in disease prevalence and in the number of deaths. The smoking cessation scenario results in the next largest decrease. The reduction of smoking prevalence under the smoking initiation scenario builds up over time and will be highly effective in the future, while being least effective in the short run. Of course, the potential effects of the population-wide policy are the largest as this scenario, by definition, reaches the entire population, whereas the other scenarios only affect quitters or those who might take up smoking. However, given the goal of smoking eradication, it is crucial to keep the long-run benefits of the initiation intervention in mind, since here potential future smokers are kept from ever even taking up the habit.

The fact that the population-wide policy yields larger effects may be seen as support for Rose's claim that population strategies are often the most effective, in contrast to the cessation intervention which could be classified as a “high risk approach” according to his classification [7]. However, the gains of the population-wide scenario presented here can only be realized if sufficient smoking cessation services are available that enable smokers to successfully quit smoking.

The effects of the future reduction in smoking prevalence have implications in terms of health. All three intervention scenarios resulted in fewer excess prevalence cases of smoking-related diseases such as COPD, lung cancer and ischemic heart disease and fewer deaths after the intervention, though the level and timing of the effects differed. The population-wide smoking control policy showed the largest reduction in disease prevalence, followed by the smoking cessation scenario. On the other hand, we see virtually no effect of the initiation scenario until the end of the projection interval, because the group that does not take up smoking due to the intervention, will not yet have had the chance to develop the major smoking-related diseases during most of the period, as it takes years until these adolescents enter the age ranges where incidence of these chronic diseases is substantial. In the long run the health effects of the smoking initiation scenario start to build up.

The population-wide smoking control policy also reaches the largest reduction in deaths, again followed by the cessation scenario. The effect of the population-wide smoking control policy and cessation intervention on the number of deaths first rises, then falls and in the end even completely disappears. With time the reduction in deaths due to the lower prevalence of smoking-related diseases, such as COPD, lung cancer and IHD is increasingly nullified because the intervention keeps persons alive longer, yielding an on average older population with a higher prevalence of non-smoking related diseases such as diabetes (data not shown) and dampening the reductions of prevalence of smoking-related diseases. This goes in line with estimates of (healthy) life expectancy, also calculated by the DYNAMO tool, which increase over the projection period, and where the improvement is bigger between 2010 and 2035 than between 2035 and 2060.

The differences in timing can be explained by the fact that these different types of interventions/policies target different exposure groups (current smokers vs. never/non smokers vs. entire population), and hence different age groups. For example, since the vast majority of smokers starts smoking before adulthood, interventions preventing persons from taking up the habit mainly target and affect these younger age groups. Cessation interventions, on the other hand, will mainly affect the adult population. Nationwide policy measures impacting population-wide smoking behavior such as a tax on tobacco affect both the young and the old. Age strongly affects the risks of the onset of chronic diseases, both associated and unassociated with smoking, and of death. Smoking-related diseases, such as COPD, lung cancer or IHD only start taking a substantial toll in adulthood and early old age, and within the smoking related diseases, the timing of the effects differs, partly because of variation in the incidence rates by age. Additionally, differences in timing can be explained by the effects of the interventions on mortality and hence on “surplus” aging caused by the intervention.

The dynamic modeling tool DYNAMO-HIA, with its ability to quantify the effects of interventions or policies on future risk factor prevalence and in turn on population health is a powerful instrument when the consequences of different tobacco control policies and interventions are to be assessed. Our findings not only show the different general patterns which interventions can produce but also illustrate how important it is that participation, i.e. the reach of an intervention, is as high as possible. Only then will interventions produce the desired effects on the population level. Such differences are illustrated well by our comparison of the maximum and the realistic scenario versions for the cessation and initiation interventions. This goes in line with the findings of other simulation models. Using the SIMSMOKE model it was shown that there is only a visible population effect of individual interventions if as many smokers as possible attempt to quit and as many of them also make use of the array of available quitting support tools [23], [24]. The RIVM Chronic Disease Model [25] showed comparable projections of the effects of various quit interventions on smoking prevalence in future years.

The present study modeled each intervention one by one. A combination of several interventions and policies affecting different target groups and covering different time horizons will yield better tobacco control outcomes than the implementation of just one intervention quantified in this study. However, in the situation of more interventions the effect of one intervention will depend on the effect of the second intervention, and vise versa. For instance, a smoking initiation intervention that is successful in preventing adolescents from taking up smoking will reduce the potential effects of a smoking cessation intervention among adults. On the other hand, the population-wide intervention might be more effective if those who are motivated to quit because of a price increase are reached by smoking cessation interventions. These interdependencies depend on the effectiveness and reach of the interventions involved, as well as on the demographic and epidemiological context. Given our purpose to disentangle and illustrate the effects of three types of interventions, we did not model combinations of intervention types.

### Strengths and limitations

Some limitations of our analyses must be considered. While we were aiming at a realistic model, a model always remains only a simplified version of reality, here being a demonstration of stylized scenarios. Much more work can still be put into the development of actual and more elaborate scenarios. This is a simulation analysis synthesizing existing data and evidence on disease epidemiology, smoking exposure, effects of smoking exposure on diseases and effects of smoking interventions on smoking exposure, all by age and sex. DYNAMO-HIA compares the effects of interventions/policies, i.e. it quantifies a reference scenario and one or more intervention scenarios with a modified risk factor exposure. The goal is not to project future population health as such. For projecting future population health, accurate information on incidence, prevalence and excess mortality data (IPM) of the diseases included in the model are needed, while in reality those data are embedded with uncertainty. This is partly because of the presence of past trends which are not exactly known. For the DYNAMO-HIA database it was decided to include trend-free IPM data partly estimated using the DisModII software [26]. Such trend-free data are used as a neutral option, because of the lack of reliable information on trends. In view of the intended use of DYNAMO-HIA, i.e. comparing scenarios, this choice is not very significant as the same disease data are used both in the intervention and reference scenario(s). Therefore, we do not expect that this unavoidable compromise has an important effect on the outcomes of our study.

Additionally, smoking prevalence and quit and (re)start rates may be biased. Classifications of smoking exposure differ between adolescents and the adult population, as do the data sources. While for older ages non-smokers in the POLS study [9] were further distinguished into former and never smokers, below age 16 in the “Youth monitor” [10], a distinction was only made between non-smokers and smokers. A distinction of non-smokers into never and former smokers at these ages is less meaningful, as these adolescents will have smoked for only a short time anyway. Given that prevalence data of smokers from different sources did not indicate important inconsistencies, we do not expect that this has biased the results. Further, quit and (re)start rates at younger ages might be biased because at these ages flows into the non-smoking state due to quitting were not explicitly modeled, nor were restart rates. This yields an underestimation of the restart rates, but at the same time also an underestimation of the quit rates which have an opposite effect. Since the overall effect on the smoking prevalence was consistent, we do not expect bias. The only issue is that when changing the start rates in the “stop initiation scenario”, the effect may be slightly underestimated given that we used “net” start rates. However, given that the higher start rates would have been nullified by quit rates, we again expect no bias.

A second limitation relates to the translation of the effects of interventions, as reported in the literature, into parameters of a dynamic tool such as DYNAMO-HIA. For example, if intervention studies report a reduction in the prevalence of smokers, additional assumptions are needed about the origin of the reduction: less initiation of smoking, increasing the prevalence of never smokers vs. more quitting, increasing the prevalence of former smokers. This translation was needed for the population-wide smoking control policy. We made our choices explicit in the paper. Assuming that most persons start smoking up to the age of 20, it is around this age when most uncertainty exists on whether the reduction in smokers reflects less starting or more quitting. Given that future health outcomes do not differ between former and never smokers at these ages, we do not expect that this affected our estimates. For older ages, mainly the expected effect of the price increase on restart rates is embedded with uncertainty. We expected that a price increase would also reduce the likelihood that future former smokers take up smoking again, and assumed a similar decrease in restart rates as in initial start rates. But other quantifications of the effect on restart rates may be equally defendable, and might yield different changes in future smoking exposure and health. At the most extreme, assuming no change in the restart rates would imply that the effect of this intervention on smoking prevalence at adult and older ages would be virtually absent during part of the projection period. While we do not consider this a plausible scenario, it indicates that future intervention studies should also evaluate the long-term effects on future smoking behavior.

A third limitation of our study is that the comparisons of the size of the effects partly depend on the exact quantification of each intervention scenario. Given that evidence from current evaluation studies is insufficient to set all the parameters in DYNAMO-HIA or any other modeling tool with certainty, this cannot be avoided. Next to a set of maximum scenarios we presented a set of realistic scenario versions, which indicated that the general patterns remained unchanged, only showing the lesser effects due to smaller effect size and reach. Due to the model's linear behavior all specific interventions or smoking control policies with effectiveness and reach specifications in-between these two versions will produce results between the realistic and maximum variant.

The research presented here shows the general patterns of three types of smoking interventions and illustrates the general use of DYNAMO-HIA. For each of the three types of interventions a wide range of smoking control or prevention services with varying effectiveness and reach can be chosen. The effect of each specific intervention will depend on its exact specifications and the current risk factor exposure and demographic and epidemiological context, which may differ from the Dutch situation. In particular, in populations with a different smoking pattern, reflecting a different stage in the smoking epidemic and/or a different mix of smoking control policies, the room for gains that can potentially be realized by different types of interventions may differ. DYNAMO-HIA can be used to quantify these effects as it easily allows for taking such factors into account.

The key strength of our study relates to using a dynamic multistate model that distinguishes separate smoking states in order to model the effects of the different interventions/policies.

Smoking affects a large range of diseases, each to a different extent and the RRs associated with smoking also depend on age and sex. Further, the different smoking-related diseases have different epidemiological patterns (time of onset, mortality). The health effects of different types of interventions depend on the effects of the intervention on future smoking exposure at different ages. Future smoking exposure, in turn, does not only depend on prevalence at baseline, and (re)start and quit rates, but also on selective mortality, as smoking is strongly associated with mortality. Using DYNAMO-HIA allowed us to assess how an intervention affects smoking exposure in future years and in different age groups, taking into account selective mortality, and to substitute morbidity and mortality in the extra years persons are alive because of the lower mortality due to the intervention. Other models like SIMSMOKE [24] use the Potential Impact Fraction (PIF) to model the effects of interventions on transitions, and hence do not contain selective mortality.

Also, DYNAMO-HIA allowed us to take into account the effect of smoking on various diseases, each having different excess risks, which vary by age. The effect of smoking exposure on death was accounted for through the effect of smoking on incidence of the nine included diseases and through the RR of total mortality, which allows DYNAMO-HIA to take into account the effect of smoking on mortality through diseases not included in the model. Technical advantages of our software also include the fact that it requires relatively modest data input resulting in rich model output, while being freely accessible through a website.

### Conclusion

The DYNAMO-HIA model showed that all smoking interventions will be effective in the long run, the population-wide strategy being most effective in both the short and long term. The quit smoking scenario evidently will be second-most effective in the short run, though in the long run the smoking initiation scenario will be almost as effective as the smoking cessation scenario. Even if interventions aimed at preventing the initiation of smoking take a long time to become manifest in terms of health effects, they need to be part of tobacco control measures as they keep in check the numbers of new smokers. A combination of interventions and policies with different time horizons reinforcing each other would be most optimal on the way to smoking eradication.

The dynamic modeling tool DYNAMO-HIA, with its ability to quantify information on the long term impact of interventions on population health, is a powerful instrument when the consequences of different tobacco control policies and interventions are to be assessed. We can directly compare the differences in the timing as well as in the relative sizes of the effects of the scenarios.

## Supporting Information

Appendix S1 Baseline smoking prevalence and transition probabilities in the Netherlands.
(DOC)

Appendix S2 Relative risks from smoking to disease/mortality and disease prevalence at baseline.
(DOC)

Figure S1 Smoking prevalence over time; Effects of each scenario in the Netherlands (realistic version, panels a–c).
(TIF)

Figure S2 Smoking prevalence by age over time; Effects of each scenario in the Netherlands (realistic version, panels a–f).
(TIF)

Figure S3 Difference in the number of deaths; Effects of each scenario in the Netherlands (realistic version).
(TIF)

Table S1 Effects of scenarios on point prevalence of diseases in the Netherlands (realistic version).
(DOC)
