# Supplementary material for: Comparison of Tobacco Control Scenarios: Quantifying Estimates of Long-Term Health Impact Using the DYNAMO-HIA Modeling Tool
Source: PLoS One. 2012 Feb 23;7(2):e32363. doi: 10.1371/journal.pone.0032363 (PMC3285691; doi:10.1371/journal.pone.0032363)
Supplement: Appendix S1 — Baseline smoking prevalence and transition probabilities in the Netherlands. (DOC) [file pone.0032363.s001.doc]

**REFERENCE SCENARIO DATA**

Table S1.1: Baseline prevalence of smokers, former smokers and never smokers in the Netherlands in percent

|  |  | Males |  |  |  | Females |  |
| --- | --- | --- | --- | --- | --- | --- | --- |
| age | never smokers | former smokers | current smokers |  | never smokers | former smokers | current smokers |
| 0 | 100 | 0 | 0 |  | 100 | 0 | 0 |
| 1 | 100 | 0 | 0 |  | 100 | 0 | 0 |
| 2 | 100 | 0 | 0 |  | 100 | 0 | 0 |
| 3 | 100 | 0 | 0 |  | 100 | 0 | 0 |
| 4 | 100 | 0 | 0 |  | 100 | 0 | 0 |
| 5 | 100 | 0 | 0 |  | 100 | 0 | 0 |
| 6 | 100 | 0 | 0 |  | 100 | 0 | 0 |
| 7 | 100 | 0 | 0 |  | 100 | 0 | 0 |
| 8 | 100 | 0 | 0 |  | 100 | 0 | 0 |
| 9 | 100 | 0 | 0 |  | 100 | 0 | 0 |
| 10 | 100 | 0 | 0 |  | 100 | 0 | 0 |
| 11 | 98.95 | 0 | 1.05 |  | 99.05 | 0 | 0.95 |
| 12 | 97.9 | 0 | 2.1 |  | 98.1 | 0 | 1.9 |
| 13 | 89.52 | 0 | 10.48 |  | 90.48 | 0 | 9.52 |
| 14 | 78 | 0 | 22 |  | 80 | 0 | 20 |
| 15 | 72.76 | 0 | 27.24 |  | 75.24 | 0 | 24.76 |
| 16 | 58.31 | 12.24 | 29.45 |  | 57.63 | 15.62 | 26.75 |
| 17 | 53.8 | 13.62 | 32.58 |  | 54.24 | 16.58 | 29.19 |
| 18 | 49.28 | 15 | 35.72 |  | 50.84 | 17.54 | 31.62 |
| 19 | 44.76 | 16.38 | 38.85 |  | 47.44 | 18.5 | 34.06 |
| 20 | 40.25 | 17.76 | 41.99 |  | 44.04 | 19.47 | 36.49 |
| 21 | 40.08 | 18.14 | 41.77 |  | 44.18 | 19.94 | 35.88 |
| 22 | 39.92 | 18.53 | 41.55 |  | 44.32 | 20.42 | 35.26 |
| 23 | 39.76 | 18.91 | 41.34 |  | 44.46 | 20.89 | 34.65 |
| 24 | 39.59 | 19.29 | 41.12 |  | 44.6 | 21.37 | 34.04 |
| 25 | 39.43 | 19.67 | 40.9 |  | 44.74 | 21.84 | 33.42 |
| 26 | 39.26 | 20.05 | 40.68 |  | 44.87 | 22.32 | 32.81 |
| 27 | 39.1 | 20.43 | 40.46 |  | 45.01 | 22.79 | 32.2 |
| 28 | 38.94 | 20.82 | 40.25 |  | 45.15 | 23.27 | 31.58 |
| 29 | 38.77 | 21.2 | 40.03 |  | 45.29 | 23.74 | 30.97 |
| 30 | 38.61 | 21.58 | 39.81 |  | 45.43 | 24.22 | 30.35 |
| 31 | 37.72 | 22.34 | 39.94 |  | 43.97 | 25.36 | 30.68 |
| 32 | 36.83 | 23.1 | 40.07 |  | 42.51 | 26.49 | 31 |
| 33 | 35.95 | 23.85 | 40.2 |  | 41.05 | 27.63 | 31.32 |
| 34 | 35.06 | 24.61 | 40.33 |  | 39.59 | 28.77 | 31.64 |
| 35 | 34.17 | 25.37 | 40.46 |  | 38.13 | 29.91 | 31.96 |
| 36 | 33.28 | 26.13 | 40.59 |  | 36.67 | 31.05 | 32.28 |
| 37 | 32.4 | 26.89 | 40.72 |  | 35.21 | 32.18 | 32.6 |
| 38 | 31.51 | 27.65 | 40.85 |  | 33.75 | 33.32 | 32.92 |
| 39 | 30.62 | 28.4 | 40.98 |  | 32.29 | 34.46 | 33.25 |
| 40 | 29.73 | 29.16 | 41.1 |  | 30.84 | 35.6 | 33.57 |
| 41 | 28.84 | 30.65 | 40.51 |  | 30.83 | 36.14 | 33.03 |
| 42 | 27.95 | 32.13 | 39.92 |  | 30.82 | 36.68 | 32.5 |
| 43 | 27.05 | 33.61 | 39.33 |  | 30.81 | 37.23 | 31.96 |
| 44 | 26.16 | 35.09 | 38.74 |  | 30.8 | 37.77 | 31.43 |
| 45 | 25.27 | 36.58 | 38.15 |  | 30.79 | 38.32 | 30.89 |
| 46 | 24.38 | 38.06 | 37.56 |  | 30.78 | 38.86 | 30.36 |
| 47 | 23.48 | 39.54 | 36.97 |  | 30.77 | 39.4 | 29.82 |
| 48 | 22.59 | 41.02 | 36.38 |  | 30.76 | 39.95 | 29.29 |
| 49 | 21.7 | 42.51 | 35.79 |  | 30.75 | 40.49 | 28.75 |
| 50 | 20.81 | 43.99 | 35.2 |  | 30.74 | 41.04 | 28.22 |
| 51 | 20.51 | 44.81 | 34.68 |  | 31.79 | 40.82 | 27.39 |
| 52 | 20.22 | 45.63 | 34.15 |  | 32.83 | 40.61 | 26.56 |
| 53 | 19.93 | 46.45 | 33.63 |  | 33.87 | 40.39 | 25.73 |
| 54 | 19.63 | 47.27 | 33.1 |  | 34.92 | 40.18 | 24.9 |
| 55 | 19.34 | 48.09 | 32.57 |  | 35.96 | 39.96 | 24.08 |
| 56 | 19.05 | 48.91 | 32.05 |  | 37 | 39.75 | 23.25 |
| 57 | 18.75 | 49.73 | 31.52 |  | 38.05 | 39.54 | 22.42 |
| 58 | 18.46 | 50.55 | 31 |  | 39.09 | 39.32 | 21.59 |
| 59 | 18.17 | 51.36 | 30.47 |  | 40.13 | 39.11 | 20.76 |
| 60 | 17.87 | 52.18 | 29.94 |  | 41.17 | 38.89 | 19.93 |
| 61 | 16.96 | 53.83 | 29.21 |  | 42.11 | 38.28 | 19.6 |
| 62 | 16.04 | 55.48 | 28.48 |  | 43.05 | 37.67 | 19.28 |
| 63 | 15.12 | 57.13 | 27.74 |  | 43.99 | 37.07 | 18.95 |
| 64 | 14.21 | 58.78 | 27.01 |  | 44.93 | 36.46 | 18.62 |
| 65 | 13.29 | 60.43 | 26.28 |  | 45.86 | 35.85 | 18.29 |
| 66 | 12.37 | 62.08 | 25.54 |  | 46.8 | 35.24 | 17.96 |
| 67 | 11.46 | 63.73 | 24.81 |  | 47.74 | 34.63 | 17.63 |
| 68 | 10.54 | 65.38 | 24.08 |  | 48.68 | 34.02 | 17.3 |
| 69 | 9.62 | 67.03 | 23.34 |  | 49.61 | 33.41 | 16.97 |
| 70 | 8.71 | 68.68 | 22.61 |  | 50.55 | 32.8 | 16.65 |
| 71 | 8.94 | 68.72 | 22.34 |  | 51.76 | 32.43 | 15.81 |
| 72 | 9.18 | 68.75 | 22.08 |  | 52.97 | 32.05 | 14.97 |
| 73 | 9.41 | 68.78 | 21.81 |  | 54.19 | 31.68 | 14.14 |
| 74 | 9.65 | 68.82 | 21.54 |  | 55.4 | 31.3 | 13.3 |
| 75 | 9.88 | 68.85 | 21.27 |  | 56.61 | 30.93 | 12.46 |
| 76 | 10.11 | 68.88 | 21 |  | 57.82 | 30.55 | 11.62 |
| 77 | 10.35 | 68.91 | 20.74 |  | 59.03 | 30.18 | 10.79 |
| 78 | 10.58 | 68.95 | 20.47 |  | 60.24 | 29.81 | 9.95 |
| 79 | 10.82 | 68.98 | 20.2 |  | 61.45 | 29.43 | 9.11 |
| 80 | 11.05 | 69.01 | 19.93 |  | 62.67 | 29.06 | 8.28 |
| 81 | 11.25 | 69.31 | 19.43 |  | 63.53 | 28.4 | 8.06 |
| 82 | 11.45 | 69.61 | 18.94 |  | 64.4 | 27.75 | 7.85 |
| 83 | 11.65 | 69.91 | 18.44 |  | 65.27 | 27.1 | 7.64 |
| 84 | 11.84 | 70.21 | 17.95 |  | 66.13 | 26.44 | 7.42 |
| 85 | 12.04 | 70.51 | 17.45 |  | 67 | 25.79 | 7.21 |
| 86 | 12.24 | 70.81 | 16.95 |  | 67.87 | 25.14 | 6.99 |
| 87 | 12.44 | 71.11 | 16.46 |  | 68.73 | 24.49 | 6.78 |
| 88 | 12.63 | 71.41 | 15.96 |  | 69.6 | 23.83 | 6.57 |
| 89 | 12.83 | 71.71 | 15.46 |  | 70.47 | 23.18 | 6.35 |
| 90 | 13.03 | 72.01 | 14.97 |  | 71.33 | 22.53 | 6.14 |
| 91 | 13.22 | 72.31 | 14.47 |  | 72.2 | 21.88 | 5.92 |
| 92 | 13.42 | 72.61 | 13.97 |  | 73.07 | 21.22 | 5.71 |
| 93 | 13.62 | 72.9 | 13.48 |  | 73.93 | 20.57 | 5.5 |
| 94 | 13.82 | 73.2 | 12.98 |  | 74.8 | 19.92 | 5.28 |
| 95 | 14.01 | 73.5 | 12.48 |  | 75.67 | 19.26 | 5.07 |

Source: Based on [9] and [10] For further information please refer to the data documentation section of the

DYNAMO-HIA project website: www.dynamo-hia.eu

Table S1.2: Baseline smoking (re)start and quit transition probabilities in the Netherlands

|  |  | Males |  |  |  | Females |  |
| --- | --- | --- | --- | --- | --- | --- | --- |
| age | start transition probability | quit transition probability | restart transition probability |  | start transition probability | quit transition probability | restart transition probability |
| 0 | 0 | 0 | 0 |  | 0 | 0 | 0 |
| 1 | 0 | 0 | 0 |  | 0 | 0 | 0 |
| 2 | 0 | 0 | 0 |  | 0 | 0 | 0 |
| 3 | 0 | 0 | 0 |  | 0 | 0 | 0 |
| 4 | 0 | 0 | 0 |  | 0 | 0 | 0 |
| 5 | 0 | 0 | 0 |  | 0 | 0 | 0 |
| 6 | 0 | 0 | 0 |  | 0 | 0 | 0 |
| 7 | 0 | 0 | 0 |  | 0 | 0 | 0 |
| 8 | 0 | 0 | 0 |  | 0 | 0 | 0 |
| 9 | 0 | 0 | 0 |  | 0 | 0 | 0 |
| 10 | 0.0105 | 0 | 0 |  | 0.0095 | 0 | 0 |
| 11 | 0.0106 | 0 | 0 |  | 0.0096 | 0 | 0 |
| 12 | 0.0856 | 0 | 0 |  | 0.0777 | 0 | 0 |
| 13 | 0.1287 | 0 | 0 |  | 0.1158 | 0 | 0 |
| 14 | 0.0672 | 0 | 0 |  | 0.0595 | 0 | 0 |
| 15 | 0.0303 | 0 | 0 |  | 0.0264 | 0 | 0 |
| 16 | 0.054 | 0.0332 | 0.3927 |  | 0.0545 | 0.0666 | 0.3426 |
| 17 | 0.0549 | 0.0409 | 0.4294 |  | 0.0537 | 0.079 | 0.3827 |
| 18 | 0.0529 | 0.0477 | 0.4242 |  | 0.0506 | 0.0878 | 0.3907 |
| 19 | 0.0487 | 0.0537 | 0.3882 |  | 0.0458 | 0.0937 | 0.3741 |
| 20 | 0.0431 | 0.059 | 0.3326 |  | 0.0398 | 0.0974 | 0.3402 |
| 21 | 0.0367 | 0.0635 | 0.2688 |  | 0.0334 | 0.0997 | 0.2963 |
| 22 | 0.0303 | 0.0674 | 0.2079 |  | 0.0271 | 0.1011 | 0.25 |
| 23 | 0.0244 | 0.0707 | 0.1612 |  | 0.0216 | 0.1024 | 0.2085 |
| 24 | 0.0192 | 0.0734 | 0.1268 |  | 0.0167 | 0.1036 | 0.1721 |
| 25 | 0.0146 | 0.0754 | 0.1027 |  | 0.0126 | 0.1044 | 0.1409 |
| 26 | 0.0108 | 0.0769 | 0.0869 |  | 0.0092 | 0.1047 | 0.1151 |
| 27 | 0.0077 | 0.0776 | 0.0776 |  | 0.0064 | 0.1045 | 0.095 |
| 28 | 0.0054 | 0.0777 | 0.0728 |  | 0.0043 | 0.1036 | 0.0807 |
| 29 | 0.0037 | 0.0772 | 0.0714 |  | 0.0028 | 0.102 | 0.0712 |
| 30 | 0.0027 | 0.0762 | 0.0724 |  | 0.0017 | 0.0996 | 0.0655 |
| 31 | 0.002 | 0.0746 | 0.0747 |  | 0.001 | 0.0966 | 0.0626 |
| 32 | 0.0017 | 0.0726 | 0.0771 |  | 0 | 0.0927 | 0.0615 |
| 33 | 0.0017 | 0.0702 | 0.0787 |  | 0 | 0.0881 | 0.0611 |
| 34 | 0.0018 | 0.0675 | 0.0794 |  | 0 | 0.083 | 0.0611 |
| 35 | 0.002 | 0.0647 | 0.079 |  | 0 | 0.0775 | 0.0613 |
| 36 | 0.0024 | 0.062 | 0.0775 |  | 0 | 0.072 | 0.0613 |
| 37 | 0.0027 | 0.0595 | 0.0748 |  | 0 | 0.0667 | 0.0609 |
| 38 | 0.003 | 0.0573 | 0.0707 |  | 0.0013 | 0.0618 | 0.0596 |
| 39 | 0.0033 | 0.0555 | 0.0657 |  | 0.0017 | 0.0573 | 0.0577 |
| 40 | 0.0035 | 0.0541 | 0.0601 |  | 0.0021 | 0.0535 | 0.0552 |
| 41 | 0.0038 | 0.0532 | 0.0542 |  | 0.0025 | 0.0504 | 0.0523 |
| 42 | 0.004 | 0.0528 | 0.0484 |  | 0.0029 | 0.0481 | 0.049 |
| 43 | 0.0042 | 0.0529 | 0.043 |  | 0.0032 | 0.0467 | 0.0456 |
| 44 | 0.0044 | 0.0534 | 0.0382 |  | 0.0035 | 0.0461 | 0.042 |
| 45 | 0.0047 | 0.0542 | 0.034 |  | 0.0037 | 0.0461 | 0.0384 |
| 46 | 0.0049 | 0.0553 | 0.0306 |  | 0.0038 | 0.0467 | 0.0349 |
| 47 | 0.0052 | 0.0564 | 0.0278 |  | 0.0039 | 0.0477 | 0.0317 |
| 48 | 0.0056 | 0.0576 | 0.0259 |  | 0.0038 | 0.049 | 0.0288 |
| 49 | 0.006 | 0.0587 | 0.0246 |  | 0.0037 | 0.0505 | 0.0262 |
| 50 | 0.0063 | 0.0598 | 0.0236 |  | 0.0035 | 0.0521 | 0.0238 |
| 51 | 0.0066 | 0.0608 | 0.0226 |  | 0.0033 | 0.0539 | 0.0218 |
| 52 | 0.0068 | 0.0619 | 0.0212 |  | 0.0031 | 0.0556 | 0.0199 |
| 53 | 0.0068 | 0.063 | 0.0193 |  | 0.0029 | 0.0574 | 0.0182 |
| 54 | 0.0067 | 0.0641 | 0.0171 |  | 0.0026 | 0.0591 | 0.0166 |
| 55 | 0.0065 | 0.0652 | 0.0148 |  | 0.0024 | 0.0607 | 0.0153 |
| 56 | 0.0062 | 0.0664 | 0.0128 |  | 0.0022 | 0.0624 | 0.0141 |
| 57 | 0.0058 | 0.0676 | 0.0112 |  | 0.002 | 0.064 | 0.0131 |
| 58 | 0.0053 | 0.0689 | 0.0105 |  | 0.0018 | 0.0656 | 0.0122 |
| 59 | 0.0048 | 0.0702 | 0.0102 |  | 0.0016 | 0.0672 | 0.0115 |
| 60 | 0.0043 | 0.0715 | 0.0104 |  | 0.0014 | 0.0688 | 0.0109 |
| 61 | 0.0038 | 0.0725 | 0.0108 |  | 0.0012 | 0.0704 | 0.0105 |
| 62 | 0.0034 | 0.0732 | 0.0112 |  | 0 | 0.072 | 0.0101 |
| 63 | 0.0031 | 0.0734 | 0.0114 |  | 0 | 0.0736 | 0.0098 |
| 64 | 0.003 | 0.0734 | 0.0115 |  | 0 | 0.0752 | 0.0095 |
| 65 | 0.0029 | 0.0731 | 0.0114 |  | 0 | 0.0768 | 0.0093 |
| 66 | 0.0029 | 0.0726 | 0.011 |  | 0 | 0.0784 | 0.009 |
| 67 | 0.0028 | 0.072 | 0.0105 |  | 0 | 0.08 | 0.0087 |
| 68 | 0.0027 | 0.0713 | 0.0096 |  | 0 | 0.0816 | 0.0083 |
| 69 | 0.0026 | 0.0706 | 0.0086 |  | 0 | 0.0832 | 0.0078 |
| 70 | 0.0024 | 0.0701 | 0.0076 |  | 0 | 0.0848 | 0.0072 |
| 71 | 0.0022 | 0.0697 | 0.0065 |  | 0 | 0.0864 | 0.0067 |
| 72 | 0.002 | 0.0695 | 0.0055 |  | 0 | 0.088 | 0.0062 |
| 73 | 0.0018 | 0.0697 | 0.0047 |  | 0 | 0.0896 | 0.0057 |
| 74 | 0.0016 | 0.0702 | 0.0041 |  | 0 | 0.0912 | 0.0052 |
| 75 | 0.0014 | 0.0709 | 0.0036 |  | 0 | 0.0928 | 0.0048 |
| 76 | 0.0012 | 0.0719 | 0.0033 |  | 0 | 0.0945 | 0.0044 |
| 77 | 0 | 0.0732 | 0.003 |  | 0 | 0.0961 | 0.004 |
| 78 | 0 | 0.0746 | 0.0029 |  | 0 | 0.0978 | 0.0036 |
| 79 | 0 | 0.0761 | 0.0028 |  | 0 | 0.0994 | 0.0031 |
| 80 | 0 | 0.0777 | 0.0028 |  | 0 | 0.101 | 0.0027 |
| 81 | 0 | 0.0793 | 0.0028 |  | 0 | 0.1026 | 0.0023 |
| 82 | 0 | 0.0809 | 0.0029 |  | 0 | 0.1041 | 0.0019 |
| 83 | 0 | 0.0824 | 0.0029 |  | 0 | 0.1055 | 0.0015 |
| 84 | 0 | 0.0837 | 0.003 |  | 0 | 0.1068 | 0.0011 |
| 85 | 0 | 0.0849 | 0.003 |  | 0 | 0.1079 | 0 |
| 86 | 0 | 0.0858 | 0.003 |  | 0 | 0.1088 | 0 |
| 87 | 0 | 0.0866 | 0.003 |  | 0 | 0.1096 | 0 |
| 88 | 0 | 0.087 | 0.003 |  | 0 | 0.11 | 0 |
| 89 | 0 | 0.0873 | 0.003 |  | 0 | 0.1103 | 0 |
| 90 | 0 | 0.0874 | 0.003 |  | 0 | 0.1103 | 0 |
| 91 | 0 | 0.0873 | 0.003 |  | 0 | 0.1103 | 0 |
| 92 | 0 | 0.0873 | 0.003 |  | 0 | 0.1103 | 0 |
| 93 | 0 | 0.0872 | 0.003 |  | 0 | 0.1102 | 0 |
| 94 | 0 | 0.0871 | 0.003 |  | 0 | 0.1101 | 0 |
| 95 | 0 | 0.0871 | 0.003 |  | 0 | 0.1101 | 0 |

Source: Based on [11] and [10]
