# Supplementary material for: Comparison of Tobacco Control Scenarios: Quantifying Estimates of Long-Term Health Impact Using the DYNAMO-HIA Modeling Tool
Source: PLoS One. 2012 Feb 23;7(2):e32363. doi: 10.1371/journal.pone.0032363 (PMC3285691; doi:10.1371/journal.pone.0032363)
Supplement: Appendix S2 — Relative risks from smoking to disease/mortality and disease prevalence at baseline. (DOC) [file pone.0032363.s002.doc]

**RELATIVE RISKS**

Table S2.1: Relative risks from smoking categories to disease and all-cause mortality

| Outcome | Males aged 35 years and over | | |  | Females aged 35 years and over | | |
| --- | --- | --- | --- | --- | --- | --- | --- |
|  | Smoking categories | | |  | Smoking categories | | |
|  | never | current | former |  | never | current | former |
| All-cause mortality: |  |  |  |  |  |  |  |
| Persons Aged 35-39 | 1 | 2.07 | * |  | 1 | 1.74 | * |
| Persons Aged 40-44 | 1 | 2.07 | * |  | 1 | 1.74 | * |
| Persons Aged 45-49 | 1 | 2.07 | * |  | 1 | 1.74 | * |
| Persons Aged 50-54 | 1 | 2.07 | * |  | 1 | 1.74 | * |
| Persons Aged 55-59 | 1 | 2.07 | * |  | 1 | 1.74 | * |
| Persons Aged 85-95 | 1 | 2.07 | * |  | 1 | 1.74 | * |
| Persons Aged 85-95 | 1 | 2.07 | * |  | 1 | 1.74 | * |
| IHD, Persons Aged 35-64 | 1 | 2.8 | * |  | 1 | 3.08 | * |
| IHD, Persons Ages 65+ | 1 | 1.51 | * |  | 1 | 1.6 | * |
| Stroke, Persons Ages 35-64 | 1 | 3.27 | * |  | 1 | 4 | * |
| Stroke, Persons Ages 65+ | 1 | 1.63 | * |  | 1 | 1.49 | * |
| Diabetes mellitus | 1 | 1 | * |  | 1 | 1 | * |
| COPD | 1 | 10.58 | * |  | 1 | 13.08 | * |
| Lung cancer | 1 | 23.26 | * |  | 1 | 12.69 | * |
| Colorectal cancer | 1 | 1 | * |  | 1 | 1 | * |
| Oral cancer | 1 | 10.89 | * |  | 1 | 5.08 | * |
| Breast cancer | - | - | - |  | 1 | 1 | * |
| Esophageal cancer | 1 | 6.67 | * |  | 1 | 7.75 | * |

Source: DYNAMO-HIA database (American Cancer Society´s Cancer Prevention Study II age-specific relative risks, 1982-1988). For further information please refer to the data documentation section of the DYNAMO-HIA project website: www.dynamo-hia.eu

* For former smokers the relative risk is equal to the relative risk for current smokers at the moment of quitting, and afterwards declines towards1. The rate of decline is similar to that described in: Hoogenveen RT, van Baal PH, Boshuizen HC, Feenstra TL., Dynamic effects of smoking cessation on disease incidence, mortality and quality of life: The role of time since cessation. Cost Eff Resour Alloc. 2008 Jan 11;6:1.

**AGE-SPECIFIC DISEASE PREVALENCES**

Table S2.2: Baseline age-specific disease prevalences in the Netherlands

|  |  | Age Range | Breast Cancer | Colorectal Cancer | COPD | Diabetes | Esophageal Cancer | IHD | Lung Cancer | Oral Cancer | Stroke |
| --- | --- | --- | --- | --- | --- | --- | --- | --- | --- | --- | --- |
| Netherlands | males | (0,15] | - | 0.00% | 0.00% | 0.00% | 0.00% | 0.00% | 0.00% | 0.00% | 0.00% |
|  |  | (15,30] | - | 0.00% | 0.00% | 0.00% | 0.00% | 0.00% | 0.00% | 0.00% | 0.00% |
|  |  | (30,45] | - | 0.00% | 0.10% | 1.30% | 0.00% | 0.30% | 0.00% | 0.00% | 0.10% |
|  |  | (45,60] | - | 0.30% | 1.50% | 5.70% | 0.00% | 3.50% | 0.10% | 0.20% | 0.90% |
|  |  | (60,75] | - | 1.70% | 5.00% | 12.70% | 0.10% | 13.60% | 0.40% | 0.50% | 4.50% |
|  |  | (75,95] | - | 4.10% | 6.60% | 15.90% | 0.10% | 28.20% | 0.80% | 0.90% | 12.60% |
|  | females | (0,15] | 0.00% | 0.00% | 0.00% | 0.00% | 0.00% | 0.00% | 0.00% | 0.00% | 0.00% |
|  |  | (15,30] | 0.00% | 0.00% | 0.00% | 0.00% | 0.00% | 0.00% | 0.00% | 0.00% | 0.00% |
|  |  | (30,45] | 0.50% | 0.00% | 0.10% | 1.00% | 0.00% | 0.10% | 0.00% | 0.00% | 0.10% |
|  |  | (45,60] | 2.60% | 0.30% | 1.90% | 4.20% | 0.00% | 1.60% | 0.10% | 0.10% | 0.70% |
|  |  | (60,75] | 5.70% | 1.30% | 4.80% | 11.70% | 0.00% | 7.80% | 0.20% | 0.30% | 3.50% |
|  |  | (75,95] | 8.60% | 3.00% | 5.30% | 17.40% | 0.00% | 18.90% | 0.20% | 0.40% | 10.10% |

Source: own calculations based on DYNAMO-HIA database. For further information please refer to the data documentation section of the DYNAMO-HIA project website: www.dynamo-hia.eu
