# Supplementary figures and images for: Comparison of Tobacco Control Scenarios: Quantifying Estimates of Long-Term Health Impact Using the DYNAMO-HIA Modeling Tool
Source: PLoS One. 2012 Feb 23;7(2):e32363. doi: 10.1371/journal.pone.0032363 (PMC3285691; doi:10.1371/journal.pone.0032363)

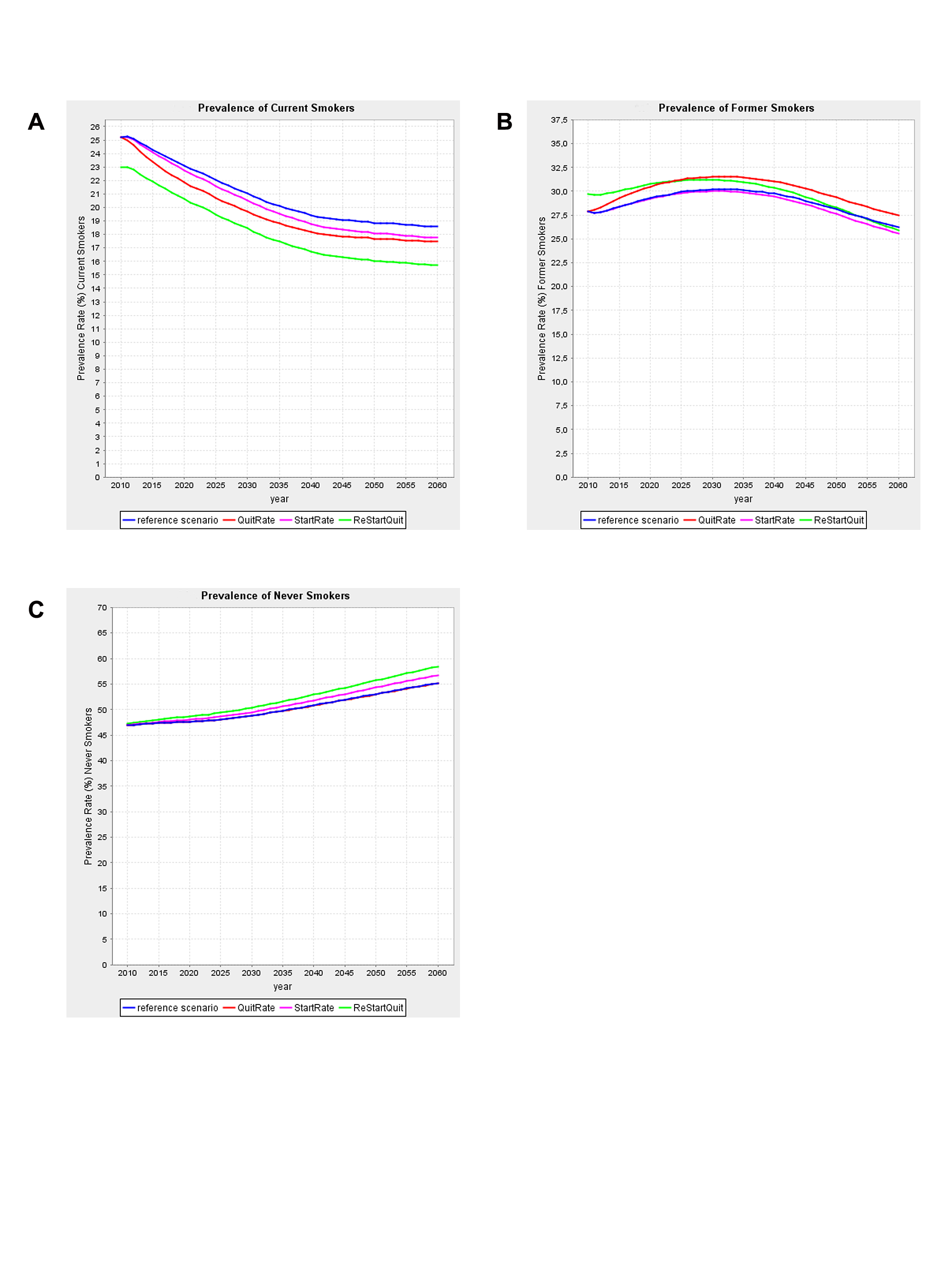

Supplement: Figure S1 — Smoking prevalence over time; Effects of each scenario in the Netherlands (realistic version, panels a–c). (TIF) [file pone.0032363.s003.tif]

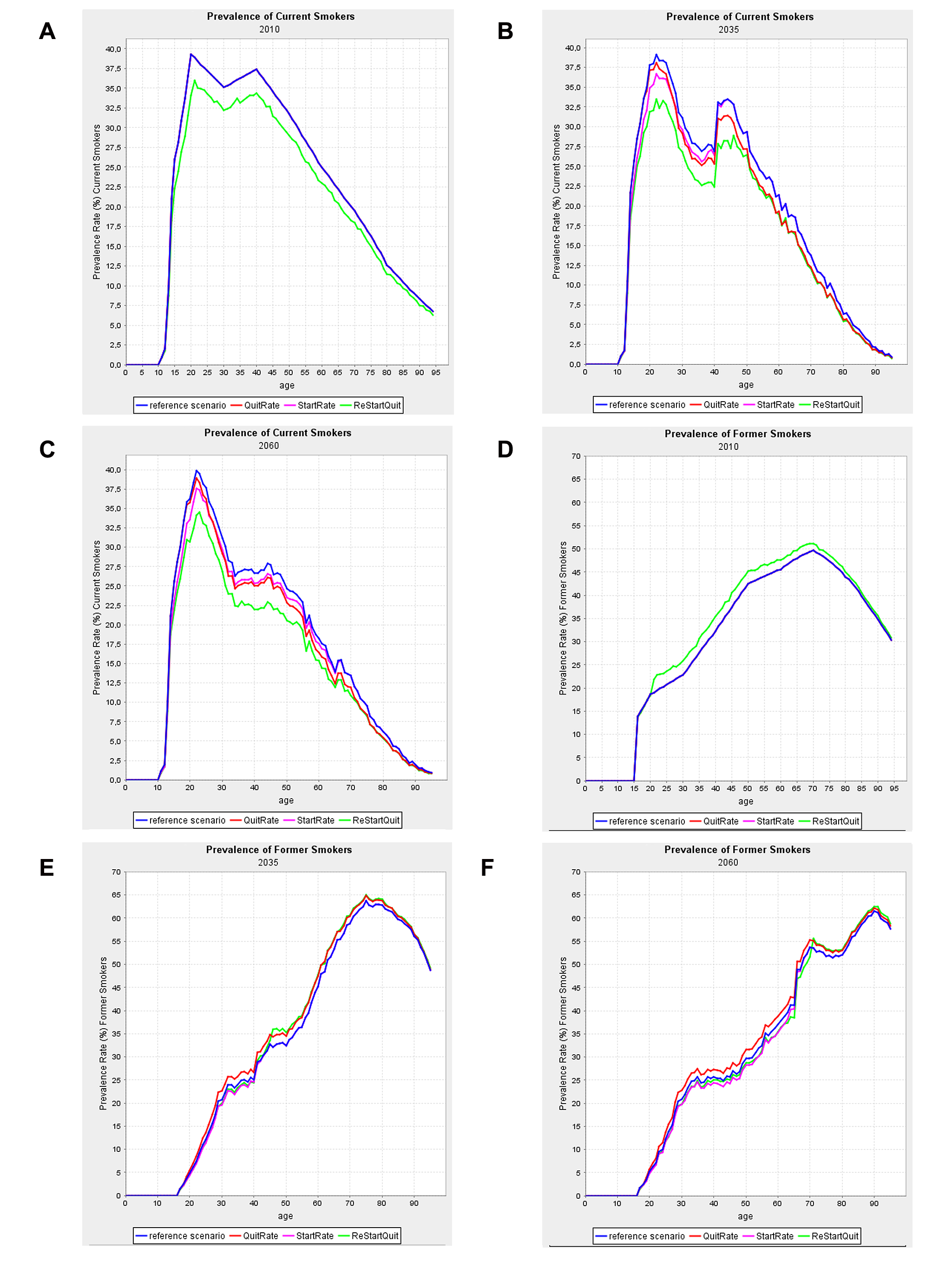

Supplement: Figure S2 — Smoking prevalence by age over time; Effects of each scenario in the Netherlands (realistic version, panels a–f). (TIF) [file pone.0032363.s004.tif]

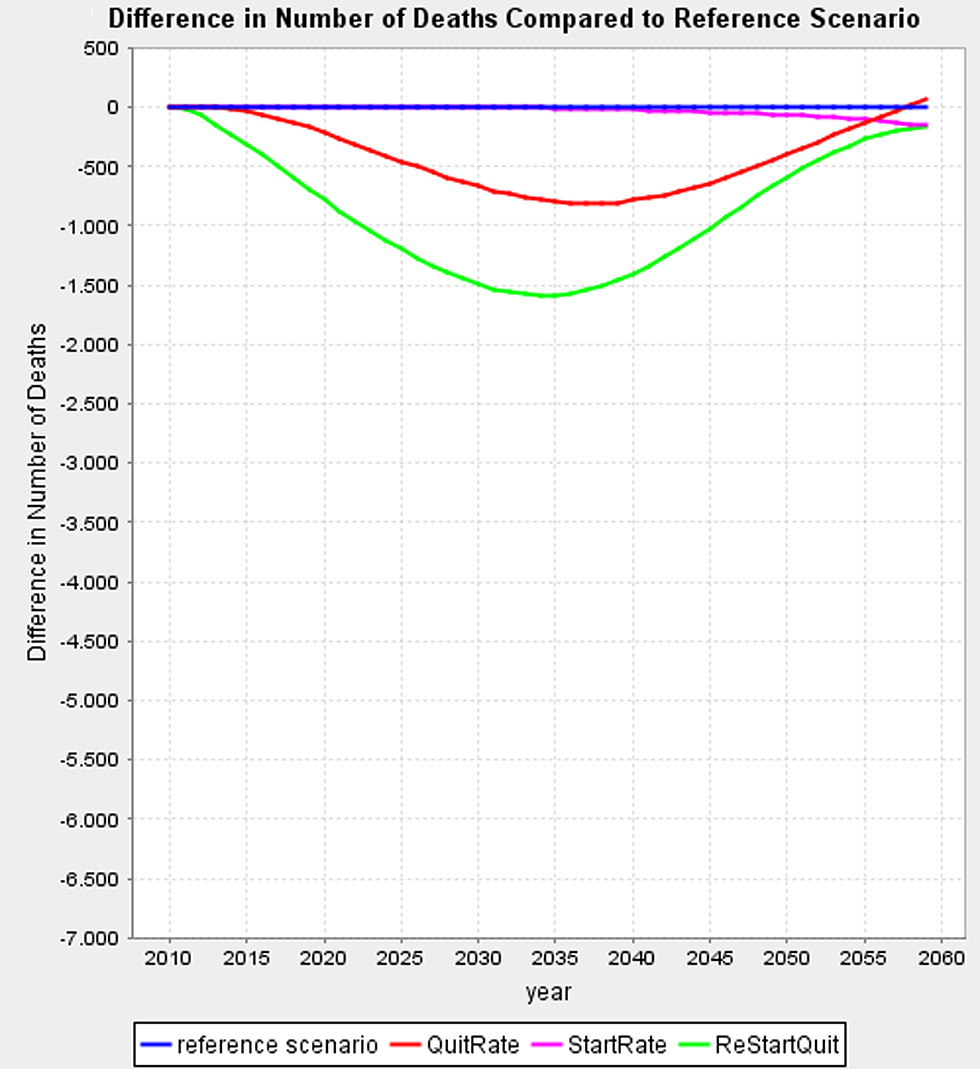

Supplement: Figure S3 — Difference in the number of deaths; Effects of each scenario in the Netherlands (realistic version). (TIF) [file pone.0032363.s005.tif]
