# Supplementary material for: Comparison of Tobacco Control Scenarios: Quantifying Estimates of Long-Term Health Impact Using the DYNAMO-HIA Modeling Tool
Source: PLoS One. 2012 Feb 23;7(2):e32363. doi: 10.1371/journal.pone.0032363 (PMC3285691; doi:10.1371/journal.pone.0032363)
Supplement: Table S1 — Effects of scenarios on point prevalence of diseases in the Netherlands (realistic version). (DOC) [file pone.0032363.s006.doc]

Table S1: Effects of scenarios on point prevalence of diseases in the Netherlands (realistic version)

|  | Absolute Level and Reduction in Disease Prevalence as Compared to Reference Scenario | | | | | | | | | |
| --- | --- | --- | --- | --- | --- | --- | --- | --- | --- | --- |
|  | 2010-2035 | | | |  |  | 2010-2060 | | | |
|  | Lung Cancer | COPD | IHD | at least one disease |  |  | Lung Cancer | COPD | IHD | at least one disease |
| Absolute Baseline Prevalence 2010 | 12,863 | 211,798 | 508,596 | 1,483,769 |  |  | 12,863 | 211,798 | 508,596 | 1,483,769 |
| change Scenario 1 (Cessation) | 591 | 7,217 | 4,793 | 9,542 |  |  | 551 | 7,860 | 4,737 | 6,525 |
| change Scenario 2 (Initiation) | 1 | 0 | 19 | 23 |  |  | 101 | 1,159 | 2,064 | 3,304 |
| change Scenario 3 (Population-Wide Policy) | 1,149 | 15,076 | 11,744 | 20,263 |  |  | 1,236 | 17,796 | 15,691 | 20,980 |
|  |  | | | | | | | | | |
|  | Percentage and Reduction in Disease Prevalence as Compared to Reference Scenario in Percentage Points | | | | | | | | | |
|  | 2010-2035 | | | |  | | 2010-2060 | | | |
|  | Lung Cancer | COPD | IHD | at least one disease |  | | Lung Cancer | COPD | IHD | at least one disease |
| Baseline Prevalence 2010 in Percent | 0.079 | 1.305 | 3.130 | 9.131 |  |  | 0.079 | 1.305 | 3.130 | 9.131 |
| change Scenario 1 (Cessation) | 0.004 | 0.044 | 0.031 | 0.063 |  | | 0.004 | 0.052 | 0.036 | 0.058 |
| change Scenario 2 (Initiation) | 0.000 | 0.000 | 0.000 | 0.000 |  | | 0.001 | 0.008 | 0.014 | 0.022 |
| change Scenario 3 (Population-Wide Policy) | 0.007 | 0.092 | 0.076 | 0.137 |  | | 0.008 | 0.118 | 0.112 | 0.168 |

*out of: diabetes, ischemic heart disease, stroke, lung cancer, oral cancer, esophageal cancer, colorectal cancer, breast cancer, COPD
